# Predictors of vitamin D deficiency and quality of life in obese patients with obstructive sleep apnea

**DOI:** 10.1038/s41598-026-36267-x

**Published:** 2026-01-20

**Authors:** Huai Heng Loh, Siow Phing Tay, Ai Jiun Koa, Mei Ching Yong, Asri Said, Chee Shee Chai, Natasya Marliana Abdul Malik, Anselm Ting Su, Bonnie Bao Chee Tang, Florence Hui Sieng Tan, Norlela Sukor

**Affiliations:** 1 Department of Medicine, Faculty of Medicine, Universiti Kebangsaan Malaysia, Kuala Lumpur, Malaysia; 2 Faculty of Medicine and Health Sciences, Universiti Malaysia Sarawak, Kota Samarahan, Sarawak Malaysia; 3 Department of Medicine, Sarawak General Hospital, Ministry of Health, Kuching, Sarawak Malaysia; 4 Sunway Medical Center Damansara, Petaling Jaya, Selangor Malaysia; 5 Department of Medicine, Hospital Canselor Tuanku Muhriz, Kuala Lumpur, Malaysia

**Keywords:** 25-Hydroxyvitamin D, Vitamin D deficiency, Sleep disorders, WHOQOL-BREF, Endocrinology, Health care, Medical research, Risk factors

## Abstract

Obstructive sleep apnea (OSA) and obesity are risk factors for adverse cardiovascular outcomes. Vitamin D deficiency (VDD) is linked to metabolic dysfunctions, contributing to cardiovascular disease. In this cross-sectional study, we aimed to determine the vitamin D profile, evaluate the impact of vitamin D level on quality of life (QoL), and identify factors associated with hypovitaminosis D in obese patients with OSA. Anthropometric data, QoL scores, and blood for serum 25-hydroxyvitamin D [25(OH)D] and metabolic parameters were collected from patients who fulfilled study criteria. There was a high prevalence of VDD and insufficiency in the study cohort, but no difference in serum 25(OH)D level across the OSA categories. Serum 25(OH)D level was associated with gender, smoking, education status, body mass index (BMI), and triglyceride level. QoL scores were not different across the vitamin D categories but were lower in severe OSA compared to mild and moderate disease. This is the first study reporting factors associated with hypovitaminosis D among OSA patients in a Southeast Asian country and its effect on their QoL. Those with VDD were more likely to be females, smokers, have received higher education, with higher BMI and triglyceride levels. The OSA severity and QoL of these patients were not affected by vitamin D levels.

**Supplementary Information:**

The online version contains supplementary material available at 10.1038/s41598-026-36267-x.

## Introduction

Obstructive sleep apnea (OSA) is a common sleep-related breathing disorder, marked by recurring episodes of partial or complete upper airway occlusion, leading to disrupted airflow^1^. This results in intermittent drops in oxygen levels and fragmented sleep, contributing to poor sleep quality^2^. Due to the rise in obesity rate, the prevalence of OSA has rapidly increased worldwide^3^, yet is widely underdiagnosed^4^. OSA is associated with heightened cardiovascular risks and is reported to be an independent risk factor for adverse cardiovascular outcomes^5,6^. In addition, untreated OSA has been shown to significantly reduce quality of life (QoL)^7,8^.

Recent literature suggests a bi-directional relationship between vitamin D deficiency (VDD) and OSA^9^. On one hand, OSA may contribute to VDD through reduced sunlight exposure, obesity-related vitamin D sequestration, autonomic dysregulation, and hypoxia-inducible factor 1-α mechanisms. Conversely, VDD may exacerbate OSA via vitamin D receptor gene polymorphisms, impaired skeletal muscle function, and increased pro-inflammatory cytokine activity. These emerging insights suggest that vitamin D status may be an important, yet underexplored, factor in the interplay between obesity and OSA.Vitamin D is mostly obtained from cutaneous synthesis with sunlight exposure and to a lesser amount through dietary sources^10^. Being lipophilic, vitamin D is stored in the adipose tissue, leading to inadequate vitamin D availability in the blood among obese individuals^11^. Hence, VDD is closely related to obesity^12^.

Traditionally, vitamin D is considered essential primarily for maintaining calcium balance and bone health. In the last decade, however, there have been rising interests in the role of vitamin D in extra-skeletal associations, particularly cardio-metabolic complications^13^. VDD has been reported to be linked to elevated risks of metabolic dysfunctions, including dyslipidemia, hypertension, and impaired glucose homeostasis, which contribute to cardiovascular disease^14^. However, the specific clinical and biochemical characteristics that predispose certain individuals with OSA and obesity to low vitamin D levels remain unclear, warranting further investigation to identify associated factors.

Malaysia, despite being a tropical country located at the Equator and sunny all year round, records a high prevalence of VDD and vitamin D insufficiency (VDI) in otherwise healthy individuals, especially among females, those living in the urban areas, and Malay and Indian ethnicities^15^. Similar patterns have been reported in other Southeast Asia countries including Singapore, Thailand, and Vietnam^16–18^, underscoring that hypovitaminosis D is widespread in this region despite abundant sunlight.

Meta-analyses have consistently demonstrated low vitamin D levels among patients with OSA^19–22^, particularly females, older patients, African American origin, higher body mass index (BMI) and larger waist circumference, and moderate to severe disease^19,23,24^. Furthermore, VDD has been associated with poor sleep health, causing daytime somnolence and poor sleep quality^25^, strengthening the association between hypovitaminosis D with sleep disorders. Nonetheless, while VDD has been well-documented in the general Malaysian and Southeast Asian population, few have examined vitamin D status specifically in patients with OSA.

Given the limited literature on the vitamin D profile of patients with OSA in Asian populations, and the uncertain relationship between metabolic parameters and hypovitaminosis D in this context, the objectives of this study were to characterize the vitamin D profile of obese OSA patients, identify clinical and biochemical parameters associated with VDD, and to evaluate the association between vitamin D status and QoL in these patients.

## Methodology

### Study design and study population

This study is part of an investigator-initiated project [*Cardiovascular Impacts of* *RAAS and Vitamin* *D* *in* *Obstructive* *Sleep* *Apnea* (*CARD-OSA*)], which examined the roles of RAAS and vitamin D in OSA and their impacts on patient health. For the CARD-OSA project, the sample size was calculated at 150 participants based on the primary outcome. While the present study addressed a secondary question, it was conducted within the same cohort.

This cross-sectional study was conducted in Sarawak General Hospital, a tertiary center, from June 2022 till April 2024. Patients who were referred for suspicion of OSA underwent sleep study. Those who fulfilled the study criteria were invited to participate in this research. As Asians tend to have a higher percentage of body fat at a lower BMI compared to the Caucasians, and the risk of having cardiovascular disease is high at lower BMI than the existing BMI cut-off point, a cut-off of 27.5 kg/m^2^ was used to define obesity in our cohort of patients^26^. The inclusion criteria were age ≥ 18 years, BMI ≥ 27.5 kg/m^2^, and confirmed OSA (apnea hypopnea index, AHI ≥ 5) via polysomnography or level 3 respiratory polygraphy. Most patients underwent level 3 respiratory polygraphy in line with American Academy of Sleep Medicine (AASM) 2017 guidelines for uncomplicated adults at risk of moderate to severe OSA, whereas those with inconclusive respiratory polygraphy results or significant co-morbidities (e.g., stroke, chronic lung disease, or pulmonary hypertension) underwent polysomnography^27^. Exclusion criteria were those with (i) other sleep conditions with abnormal AHI, such as obesity hypoventilation syndrome and central sleep apnea, (ii) conditions affecting calcium or vitamin D levels, such as hyperparathyroidism, (iii) on vitamin D and calcium supplements, (iv) chronic kidney disease, (v) malignancy, and (vi) pregnancy.

Basic demographic data and anthropometric measurements, including blood pressure (BP), waist circumference, neck circumference, and BMI were collected. The BP was measured after the participants had rested for at least 5 min and was repeated after an interval of 1–2 min. The mean BP was used for analysis.

Morning blood samples were collected after an overnight fast of at least 8 h. A total of 10 ml of whole blood was drawn from the cubital vein of the participants and collected into vacutainer plain tubes for serum 25-hydroxyvitamin D [25(OH)D], intact parathyroid hormone (iPTH), corrected calcium, phosphate, and metabolic parameters including HbA1c, uric acid, and cholesterol profile. The specimen tubes were kept at room temperature and centrifuged immediately at 3000 rpm for 10 min. All the patients were administered the World Health Organization Quality of Life Brief Version (WHOQOL-BREF) questionnaire^28^. However, detailed dietary and lifestyle data of the study patients were not collected.

The control group comprised 33 healthy volunteers from the community who were non-obese, non-smokers, and free from known medical illnesses. These participants were screened through interviews, had normal Epworth Sleepiness Scale (ESS), low Berlin scores, and were not taking vitamin D or calcium supplements, chronic medications, and were not recovering from recent acute illness. Subsequently they had their mean of two BP readings documented, and weight and height measured for calculation of BMI. As a non-obese individual with normal ESS and Berlin scores had low likelihood of having OSA, sleep study was not performed in this group of healthy volunteers.

### Study variables definition

OSA was confirmed based on AASM International Classification of Sleep Disorders Third edition diagnostic criteria, and severity of OSA was categorized as mild if AHI was ≥ 5 to < 15/hour, moderate if AHI was ≥ 15 to < 30/hour, and severe if AHI was ≥ 30/hour. Hypertension was defined as systolic BP > 140 and/or diastolic BP > 90mmHg on two different days, or if patient was on anti-hypertensive medications.

Vitamin D levels were assessed as serum 25(OH)D utilizing Liaison^®^ 25 OH vitamin D total assay kit, a direct competitive chemiluminescence immunoassay (CLIA) on a fully automated chemiluminescence analyser (Liaison^®^ XL, DiaSorin, Italy). The Liaison^®^ 25 OH vitamin D total assay is a direct competitive CLIA with a measuring range of 4.0 to 150 ng/mL. Precision studies were performed in accordance with the CLSI EP15-A2 guidelines to confirm repeatability and reproducibility. Intra- and inter-assay coefficient of variations were within the manufacturer’s claims. Calibration and controls were run prior to each batch of samples to monitor assay performance. Despite the recent controversy surrounding vitamin D levels, the levels were categorized to (i) > 30 ng/mL as sufficient (VDS), (ii) 20-29.9 ng/mL as VDI and (iii) < 20ng/mL as VDD^29,30^. As Malaysia lies near the equator, with year-round sunshine, significant seasonal fluctuations in serum vitamin D levels are not expected. Therefore, the vitamin D levels observed are less likely to reflect seasonal effects.

The validated WHOQOL-BREF questionnaire consists of 26 items, with 4 domains, i.e. physical health (7 items), psychological health (6 items), social relationships (3 items), and environment health (8 items) as well as 2 questions which are examined separately: (i) the individual’s general perception of their QoL and (ii) the individual’s overall satisfaction of his or her health. The domain scores are scaled in a positive direction, i.e. higher scores denote better QoL. However, some scorings are reversed for facets which are not scaled in a positive direction. The mean score of all items within each domain is used to calculate the domain score and subsequently multiplied by 4 for a transformed 0-100 scale for comparison with the original WHOQOL-100 scores.

This study is reported in accordance with the STROBE guidelines for cross-sectional studies. Efforts to minimize bias included standardized recruitment and measurements, use of validated assays and polysomnography, and adjustment for key confounders, although residual confounding cannot be excluded.

### Statistical analysis

Statistical analysis was performed using SPSS software (version 29, SPSS Inc., Chicago, IL). Continuous variables were presented as mean ± SD if data were normally distributed, or median (25th – 75th percentile) if they were not. Categorical variables were presented as absolute count and their percentages. Variables that were normally distributed were compared among the three groups using ANOVA, and comparison of each pair with Tukey-Kramer post hoc test if ANOVA was significant. For variables that were not normally distributed, Kruskal-Wallis test was used to compare the three groups. For categorical variables, χ^2^ test was applied to test the differences between the observed frequencies of the three groups. Correlation coefficients were calculated using Pearson or Spearman’s correlation test. Multiple linear regression analysis was performed to elucidate the associated factors of hypovitaminosis D in OSA after adjustments for potential explanatory variables. A p value of < 0.05 was taken as statistical significance.

## Results

A total of 204 patients confirmed to have OSA and met the study criteria were recruited over a 22-month period (Supp Fig. 1). The baseline characteristics of these patients are presented in Supp Table. Among them, 9.3% had mild OSA, 27.9% moderate OSA, and 62.7% severe OSA. Of these patients, females represented 14 of 19 cases with mild OSA, 37 of 57 with moderate OSA, and 53 of 128 with severe OSA. There was a high prevalence of hypertension and dyslipidemia in the study population.

The cohort was subsequently divided into vitamin D categories based on their serum 25(OH)D levels (Table 1). Majority of the patients had VDD (57.8%) and VDI (34.3%). Those with VDD consisted more of females (66.9%) and from Malay ethnicity (55.9%), and majority of them were non-smokers and non-alcohol consumers. This group of patients also demonstrated higher pulse rate and BMI in comparison to patients without VDD. Otherwise, there was no significant difference in age, education status, and ESS scores. Besides, there was no difference in AHI, OSA severity, metabolic parameters, including BP, uric acid, HbA1c, and cholesterol profile, and other biochemical parameters – mean iPTH, calcium, and phosphorus levels – across the vitamin D groups. Compared to the healthy volunteers, there was a lower serum 25(OH)D and a higher prevalence of VDD among the OSA patients (Table 2). Although participants were not individually matched for age and gender, the control and OSA groups were comparable in distribution of these parameters, with no significant between-group differences (*p* > 0.05).

**Table 1:** Demographic and biochemistry data of study participants based on vitamin D statuses.

Variables	VDD, *n* = 118	VDI, *n* = 70	VDS, *n* = 16	*p*
Age, years	41.6 ± 11.7	45.4 ± 13.4	47.5 ± 9.6	0.045
Female gender, n (%)	79 (66.9)	23 (32.9)	2 (12.5)	< 0.001
**Ethnicity** Malay Chinese Bidayuh Iban Others	66 (55.9) 13 (11.0) 19 (16.1) 17 (14.4) 3 (2.5)	22 (31.4) 17 (24.3) 10 (14.3) 18 (25.7) 3 (4.3)	6 (37.5) 3 (18.8) 2 (12.5) 5 (31.3) 0 (0)	0.041
**Education, n (%)** NFE Primary Secondary Tertiary	3 (2.5) 6 (5.1) 63 (53.4) 46 (39.0)	1 (1.5) 12 (17.1) 41 (58.6) 16 (22.9)	1 (6.3) 2 (12.5) 9 (56.3) 4 (25.0)	0.064
**Smoking, n (%)** Active smoker Non-smoker Ex-smoker	10 (8.7) 82 (71.3) 23 (20.0)	17 (24.3) 33 (47.1) 20 (28.6)	3 (18.8) 5 (31.3) 8 (50.0)	< 0.001
**Alcohol intake, n (%)** Consumer Non-consumer Ex-consumer	26 (22.6) 82 (71.3) 7 (6.1)	13 (18.6) 39 (55.7) 18 (25.7)	4 (25.0) 6 (37.5) 6 (37.5)	< 0.001
AHI, per hour	45.1 ± 29.0	46.6 ± 25.7	40.9 ± 25.6	0.752
**OSA category, n (%)** Mild Moderate Severe	14 (11.9) 32 (27.1) 72 (61.0)	3 (4.2) 21 (29.2) 48 (66.7)	2 (11.8) 5 (29.4) 10 (58.8)	0.497
ESS	9.1 ± 4.9	9.7 ± 6.0	8.9 ± 4.9	0.679
SBP, mmHg	146.3 ± 19.5	145.8 ± 19.9	147.0 ± 19.2	0.739
DBP, mmHg	93.8 ± 13.7	94.6 ± 13.4	91.1 ± 13.0	0.651
Pulse rate, bpm	80.6 ± 14.5	75.2 ± 11.6*	78.4 ± 13.5	0.020
BMI, kg/m^2^	42.0 ± 7.1	39.3 ± 7.0*	36.7 ± 6.7*	0.003
iPTH, mmol/L	63.6 (44.2, 93.6)	60.9 (37.2, 78.6)	60.7 (44.3, 104.7)	0.279
25(OH)D, ng/mL	14.2 ± 3.6	24.2 ± 2.8*	34.5 ± 3.5*^#^	< 0.001
Corrected calcium, mmol/L	2.33 ± 0.10	2.32 ± 0.10	2.34 ± 0.15	0.525
Phosphate, mmol/L	1.20 ± 0.19	1.17 ± 0.22	1.17 ± 0.29	0.365
Uric acid, umol/L	413.8 ± 90.6	419.0 ± 85.4	421.1 ± 113.5	0.908
HbA1c, %	6.2 (5.8, 6.9)	6.2 (5.7, 6.8)	6.0 (5.7, 6.7)	0.780
**Lipid profile, mmol/L** Total LDL-C HDL-C Triglycerides	4.88 ± 1.11 2.84 ± 0.89 1.23 (1.11, 1.45) 1.65 (1.11, 2.28)	4.65 ± 0.94 2.74 ± 0.77 1.25 (1.13, 1.41) 1.36 (1.07, 1.82)	4.62 ± 0.89 2.71 ± 0.71 1.25 (1.18, 1.46) 1.42 (0.99, 2.03)	0.283 0.697 0.968 0.146

For group-wise comparisons across vitamin D categories, one-way ANOVA with Tukey-Kramer post hoc tests (adjusted for multiple comparisons) was applied. Significant pairwise differences are indicated by *: compared to VDD and ^#^: compared to VDI.

Numerical variables are presented as the mean ± standard deviation or median (IQR), categorical variables are defined as absolute numbers and their percentages.

VDD: vitamin D deficiency; VDI: vitamin D insufficiency; VDS: vitamin D sufficiency; NFE: no formal education; AHI: apnea hypopnea index; OSA: obstructive sleep apnea; ESS: Epworth Sleepiness Scale; SBP: systolic blood pressure; DBP: diastolic blood pressure; BMI: body mass index; iPTH: intact parathyroid hormone; 25(OH)D: 25-hydroxyvitamin D; IQR: interquartile range.

**Table 2:** Comparison of serum 25(OH)D with healthy volunteers.

Variables	OSA, *n* = 204	Healthy volunteers, *n* = 33	*p*
Age, years	43.4 ± 12.3	40.2 ± 10.2	0.170
Male gender, n (%)	100 (49.0)	21 (63.6)	0.119
ESS	9.3 ± 5.3	4.2 ± 2.4	< 0.001
Serum 25(OH)D, ng/mL	19.2 ± 7.3	23.9 ± 7.2	0.002
**Vitamin D status, n (%)** Deficiency Insufficiency Sufficiency	118 (57.8) 70 (34.3) 16 (7.8)	11 (33.3) 15 (45.5) 7 (21.2)	0.009

Numerical variables are presented as the mean ± standard deviation, categorical variables are defined as absolute numbers and their percentages.

25(OH)D: 25-hydroxyvitamin D; OSA: obstructive sleep apnea; ESS: Epworth Sleepiness Score.

Pearson’s correlation analysis demonstrated 25(OH)D to be positively correlated with age , and negatively correlated with BMI (Table 3). Gender, education status, smoking, BMI, and triglyceride levels, were significantly correlated with serum 25(OH)D in multiple linear regression analysis after controlling for confounding factors, with an adjusted R^2^ of 0.274 (Table 4). Our cohort of patients with hypovitaminosis D were more likely to be females, smokers, received secondary or tertiary education, with higher BMI and higher triglyceride level.

**Table 3:** Correlation analysis between serum 25-hydroxyvitamin D and independent variables. *Pearson’s correlation.

Variables	*r*	*p*
Age	0.190	0.007
Apnoea hypopnea index	-0.027	0.703
Systolic blood pressure	0.080	0.257
Diastolic blood pressure	0.007	0.920
Body mass index	-0.253	< 0.001
Uric acid	0.088	0.212
HbA1c	0.015	0.834
LDL-C	-0.055	0.433
HDL-C	-0.006	0.927
Triglyceride	-0.092	0.188

**Table 4:** Multiple linear regression analysis of predictors of serum 25(OH)D in patients with OSA.

Independent variable	Dependent variable: 25(OH)D Adjusted *R*^2^ = 0.274		
	β	95% CI	*p*
Age	0.109	-0.014, 0.144	0.106
Gender, female	-0.303	-6.673, -2.148	< 0.001
Ethnicity, others	0.107	0.320, 1.654	0.086
Education, secondary/tertiary	-0.138	-5.364, 0.679	0.040
Smoking	-0.178	-4.869, -0.420	0.020
Body mass index	-0.180	-0.310, -0.056	0.005
HDL-C	0.093	-0.458, 3.065	0.146
Triglyceride	-0.144	-2.319, -0.166	0.024

Controlled for alcohol consumption, AHI, SBP, DBP, uric acid, HbA1c, LDL-C.

25(OH)D: 25-hydroxyvitamin D; AHI: apnoea hypopnoea index; SBP: systolic blood pressure; DBP: diastolic blood pressure.

Patients with severe OSA demonstrated lower scores in all domains of WHOQOL-BREF except Domain 4 (environment), compared to the other two groups of OSA, particularly in the physical, psychological, and social domains (Table 5). Nevertheless, there was no difference in the QoL scores among the three vitamin D categories in all domains (Table 6).

**Table 5:** WHOQOL-BREF among OSA severity categories (transformed to 100).

	Mild, *n* = 19	Moderate, *n* = 57	Severe, *n* = 128	*p*
Quality of life perception	4.0 (3.0, 4.0)	4.0 (3.0, 4.0)	3.0 (3.0, 4.0)	0.002
Satisfaction with health	3.0 (3.0, 4.0)	3.0 (3.0, 4.0)	3.0 (2.0, 3.0)	0.027
Domain 1	63.0 (56.0, 63.0)	56.0 (44.0, 63.0)	50.0 (44.0, 56.0)	0.025
Domain 2	69.0 (56.0, 75.0)	56.0 (50.0, 69.0)	50.0 (44.0, 56.0)	< 0.001
Domain 3	75.0 (56.0, 75.0)	69.0 (56.0, 75.0)	56.0 (50.0, 75.0)	0.019
Domain 4	69.0 (63.0, 81.0)	69.0 (53.0, 81.0)	63.0 (56.0, 75.0)	0.116

**Table 6:** WHOQOL-BREF among vitamin D categories (transformed to 100).

	VDD, *n* = 118	VDI, *n* = 70	VDS, *n* = 16	*p*
Quality of life perception	4.0 (3.0, 4.0)	4.0 (3.0, 4.0)	3.0 (3.0, 4.0)	0.585
Satisfaction with health	3.0 (2.0, 3.0)	3.0 (3.0, 4.0)	3.0 (3.0, 4.0)	0.336
Domain 1	56.0 (44.0, 63.0)	56.0 (44.0, 63.0)	53.0 (44.0, 63.0)	0.704
Domain 2	56.0 (44.0, 68.0)	56.0 (50.0, 63.0)	56.0 (45.5, 69.0)	0.688
Domain 3	69.0 (50.0, 75.0)	56.0 (50.0, 75.0)	62.5 (51.5, 75.0)	0.752
Domain 4	63.0 (50.0, 75.0)	63.0 (56.0, 69.0)	63.0 (50.0, 73.5)	0.539

Variables are presented as median (IQR).

WHOQOL-BREF: World Health Organization Quality of Life Brief Version; OSA: obstructive sleep apnea; Domain 1: Physical health; Domain 2: Psychological health; Domain 3: Social relationships; Domain 4: Environmental health; VDD: vitamin D deficiency; VDI: vitamin D insufficiency; VDS: vitamin D sufficiency; IQR: interquartile range.

## Discussion

This study demonstrated three key findings. First, a high prevalence of hypovitaminosis D was observed among obese OSA patients. Second, female gender, smoking, higher education level, higher BMI, and elevated triglycerides were significantly associated with hypovitaminosis D. Last, while patients with OSA, particularly those with severe disease, reported impaired QoL, these scores were not associated with vitamin D levels.

The high prevalence of VDD in our OSA cohort is consistent with prior reports from Malaysia and other Southeast Asian populations, where hypovitaminosis D remains common despite the tropical climate^30–34^. Although plausible biological mechanisms have been proposed to link VDD with OSA pathogenesis and severity, the literature remains inconclusive. In our study, despite widespread VDD, no independent association was observed between vitamin D status and OSA severity. Instead, higher BMI emerged as a key determinant, underscoring the role of adiposity in vitamin D regulation^12^. This association is supported by studies in diverse populations, including women with osteoporosis in Thailand^35^ and healthy young adults in Australia^36^.

Several mechanisms may explain this relationship^9^. Obesity contributes to lower vitamin D levels through reduced sun exposure, volumetric dilution, and sequestration of vitamin D within adipose tissue. Altered hepatic metabolism and decreased bioavailability further exacerbate this deficiency. Together, these mechanisms account for the strong inverse association between BMI and serum 25(OH)D observed in our cohort.

Beyond obesity, our analysis identified additional associated factors of VDD. Female patients were more likely to be vitamin D deficient, consistent with prior studies across Asia and beyond^37,38^. Sociocultural factors such as sun avoidance, clothing preferences that cover most parts of the body, and application of sun block, driven in part by a preference for fairer skin, likely contributed to this observation^32,39^. Similarly, higher education was associated with greater risk of VDD probably reflecting higher likelihood of working indoors and less participation in outdoor activities, resulting in reduced sun exposure, whereas those with lower education may have greater sun exposure due to reliance on public transportation and less frequent use of sun protection^40–42^. Interestingly, the opposite association was reported in Western populations, where higher education correlated with higher vitamin D levels^38^, likely reflecting differences in lifestyle factors such as health literacy, diet, and outdoor activity. Smoking may reduce vitamin D levels by impairing cutaneous synthesis, disrupting vitamin D–PTH regulation and metabolic enzyme activity, inducing renal dysfunction through heavy metal exposure, and contributing to lower dietary intake^43^.

Although triglyceride showed no significant correlation in univariate analysis, it became significant in the multivariate model, likely reflecting confounding of BMI and gender in the simple correlation analysis. The negative association between triglycerides and vitamin D levels in our study is consistent with earlier reports from Taiwan and Korea^44,45^. Vitamin D not only regulates the expression of genes involved in lipid metabolism, hence reduces fatty acid synthesis, enhances oxidation of fatty acids and lowers triglyceride levels^46^, it also enhances intestinal calcium absorption leading to reduction in hepatic triglyceride formation and secretion^47^. Furthermore, dyslipidemia is often associated with unhealthy dietary habits that can reduce vitamin D intake and adversely influence lipid metabolism^48^.

Nevertheless, female gender and BMI remained strongly associated with vitamin D levels, while education, smoking status, and triglyceride levels demonstrated only modest associations. The p-values of these latter predictors were close to the conventional 0.05 threshold, indicating marginal statistical certainty and should therefore be interpreted with caution. Moreover, the overall model explained only about 27% of the variance in vitamin D, suggesting that other important determinants such as outdoor physical activity, sun exposure, and dietary intake may account for the remaining variability. Importantly, serum 25(OH)D levels were not correlated with other metabolic parameters, including HbA1c, uric acid, LDL-C, and HDL-C. Since glycemic control and uric acid levels are influenced by multiple factors beyond vitamin D status, this may explain the lack of association observed in our study.

In addition to metabolic markers, we also evaluated physiological mediators of vitamin D action. Neither iPTH nor corrected calcium levels differed significantly across vitamin D categories. This may reflect vitamin D sequestration in obesity and inter-individual variation in the PTH-set point, with elevations in PTH detectable only at a more severe level of deficiency. Collectively, these findings suggest that VDD in our cohort is unlikely to be primarily driven by calcium-PTH disturbances.

Despite a lower QoL being reported among patients with VDD in other disease entities^49–51^, literature is scarce on the assessment of QoL among OSA patients with low vitamin D level. Our analysis demonstrated no significant difference in the QoL scores among the three vitamin D categories, but only significantly different among the OSA severity categories, suggesting that in our patients with OSA, the QoL may be affected more by the disease severity rather than hypovitaminosis D. Nevertheless, the absence of significant QoL differences among the vitamin D groups may reflect limited statistical power due to the small sample size of vitamin D sufficient group, and unmeasured psychosocial confounders such as depression or socioeconomic status as well as obesity, OSA severity, and other co-morbidities may have exerted stronger influence on the QoL than vitamin D status alone.

### Limitations and strengths

This study has several limitations. Its cross-sectional design limits the ability to infer causality or determine the directionality of associations between VDD and its related factors. Resource constraints prevented the investigation of vitamin D-related gene polymorphisms, which may contribute to inter-individual variability in circulating 25(OH)D. In addition, we did not assess sun exposure, dietary intake, or detailed lifestyle habits, all of which could influence vitamin D status. Limited sun exposure and low dietary intake, both common in Southeast Asian populations, may have contributed to hypovitaminosis D observed in our cohort. The absence of these measures may have introduced unmeasured confounding and reduced our ability to fully explain inter-individual differences in vitamin D levels.

Although Malaysia’s equatorial location provides year-round sunlight, the influence of other unmeasured factors cannot be ruled out. Another limitation is that the control group was not objectively screened for OSA as polysomnography was not performed, although validated questionnaires were used to minimize misclassification.

Unmeasured confounders, including mood disorders and socioeconomic factors, may have influenced and affected WHOQOL-BREF scores and contributed to the non-significant QoL differences observed. The small number of vitamin D–sufficient subjects may have further limited statistical power to detect group differences. Future longitudinal studies incorporating assessments of diet, sun exposure, and physical activity are warranted to better clarify the interplay between vitamin D, obesity, dyslipidemia, and QoL.

Nevertheless, to the best of our knowledge, this is among the earliest to characterize the vitamin D profile among patients with OSA in Southeast Asia, and to examine the associated clinical parameters and their QoL in this population. Our findings extend the limited regional literature by integrating hormonal, metabolic, and psychosocial measures within the obese OSA cohort, thereby providing valuable content-specific insights into this underexplored area. These results further support the growing body of research linking OSA with vitamin D, particularly in the associated factors of hypovitaminosis D and the absence of effect of vitamin D in OSA severity and the reduced QoL in this cohort of patients.

## Conclusion

There is a high prevalence of VDI and VDD among obese OSA patients. Those with VDD were more likely to be females, smokers, have received higher education, with higher BMI and triglyceride levels, highlighting the importance of targeted screening to enable earlier detection and intervention. Beyond screening, these findings support the promotion of healthy lifestyle activities, particularly increasing safe sun exposure and physical activity among these high-risk populations. While no differences in QoL scores were found, this may reflect limited power, particularly given the small number of patients in the VDS group. Large scale interventional and prospective studies are crucial to validate these results and explore whether vitamin D repletion could modify clinical outcomes in this high-risk population to inform personalized management strategies in these patients.

## Supplementary Information

Below is the link to the electronic supplementary material.

Supplementary Material 1

Supplementary Material 2

## Data Availability

The datasets used and/or analysed during the current study are available from the corresponding author on reasonable request.

## Abbreviations

25(OH)D: 25-Hydroxyvitamin D
AHI: Apnea hypopnea index
BP: Blood pressure
ESS: Epworth Sleepiness Scale
OSA: Obstructive sleep apnea
VDD: Vitamin D deficiency
VDI: Vitamin D insufficiency
VDS: Vitamin D sufficiency
WHOQOL-BREF: World Health Organization Quality of Life Brief Version
QoL: Quality of life
