# Supplementary material for: Predictors of vitamin D deficiency and quality of life in obese patients with obstructive sleep apnea
Source: Sci Rep. 2026 Jan 20;16:5813. doi: 10.1038/s41598-026-36267-x (PMC12894656; doi:10.1038/s41598-026-36267-x)
Supplement: Supplementary file 1 — Supplementary Material 1 [file 41598_2026_36267_MOESM1_ESM.pdf]

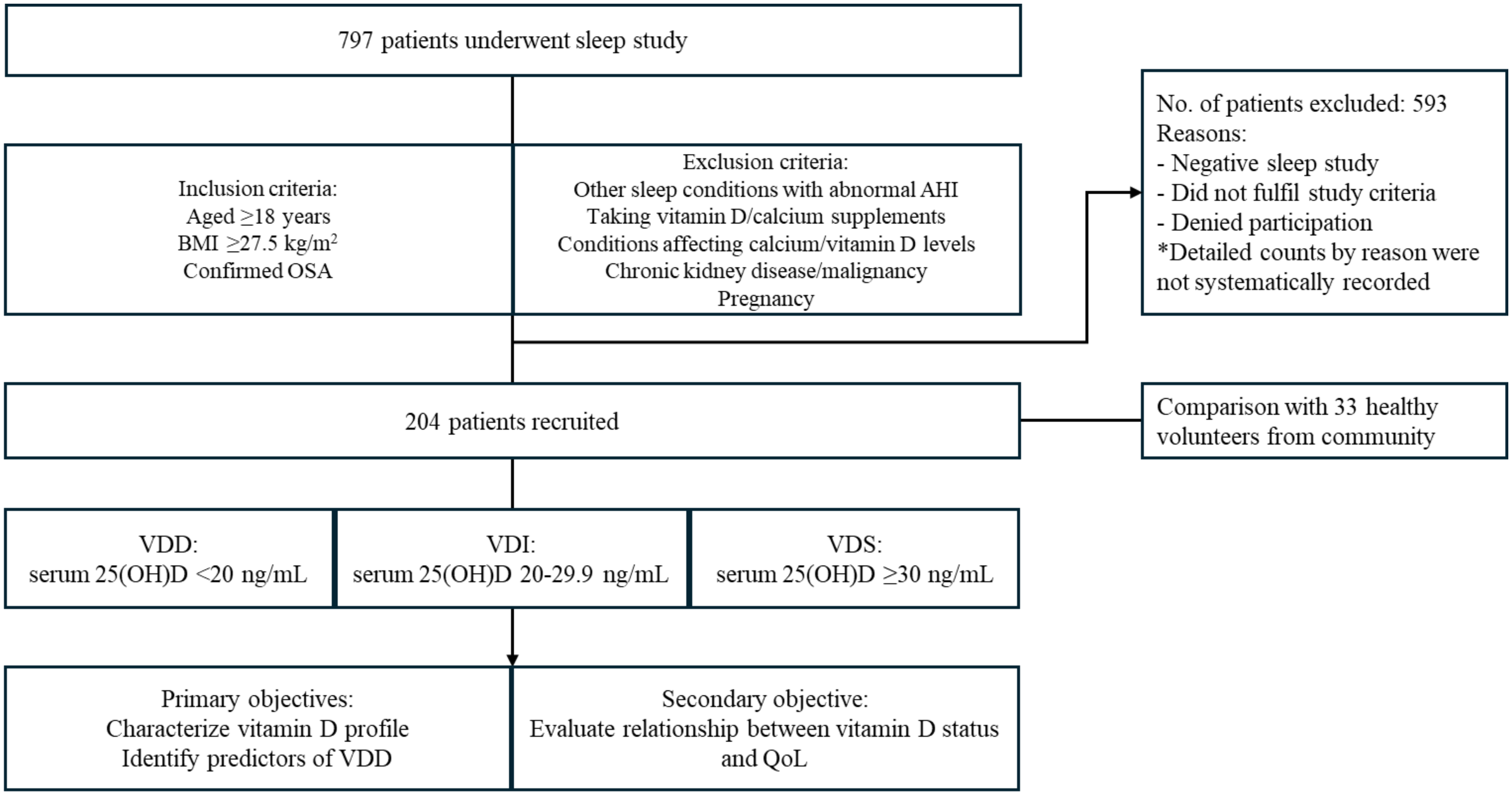

BMI: body mass index; OSA: obstructive sleep apnea; AHI: apnea hypopnea index; VDD: vitamin D deficiency; VDI: vitamin D insufficiency; VDS: vitamin D sufficiency; QoL: quality of life

Supp Fig 1 Study flowchart: patient recruitment, categorization, and objectives
