# Supplementary material for: Predictors of vitamin D deficiency and quality of life in obese patients with obstructive sleep apnea
Source: Sci Rep. 2026 Jan 20;16:5813. doi: 10.1038/s41598-026-36267-x (PMC12894656; doi:10.1038/s41598-026-36267-x)
Supplement: Supplementary file 2 — Supplementary Material 2 [file 41598_2026_36267_MOESM2_ESM.docx]

**Supp Table** Basic demography of all included patients

| **Variables** | **All patients, n=204** |
| --- | --- |
| Age, years | 43.4 ± 12.3 |
| Male gender, n (%) | 100 (49.0) |
| Ethnicity, n (%)  Malay  Chinese  Bidayuh  Iban  Others | 94 (46.1)  33 (16.2)  40 (19.6)  31 (15.2)  6 (2.9) |
| Education status, n (%)  No formal education  Primary  Secondary  Tertiary | 5 (2.5)  20 (9.8)  113 (55.4)  66 (32.4) |
| Co-morbidities, n (%)  Type 2 diabetes  Dyslipidemia  Hypertension  Hyperuricemia | 107 (52.5)  187 (91.7)  160 (78.4)  122 (59.8) |
| Smoking, n (%)  Active smoker  Non-smoker  Ex-smoker | 30 (14.9)  120 (59.7)  51 (25.4) |
| Alcohol consumption, n (%)  Yes  No  Previous | 43 (21.4)  127 (63.2)  31 (15.4) |
| ESS | 9.3 ± 5.3 |
| AHI, per hour | 39.0 (22.3, 66.0) |
| OSA severity, n (%)  Mild  Moderate  Severe | 19 (9.3)  57 (27.9)  128 (62.7) |
| SBP, mmHg | 147.0 ± 19.2 |
| DBP, mmHg | 93.9 ± 13.5 |
| Pulse rate, bpm | 78.4 ± 13.5 |
| BMI, kg/m2 | 40.7 ± 7.2 |
| Neck circumference, cm | 43.1 ± 4.1 |
| Waist circumference, cm | 118.9 ± 14.0 |
| 25(OH)D, ng/mL | 19.2 ± 7.3 |
| iPTH, mmol/L | 61.9 (43.1, 89.3) |
| Corrected calcium, mmol/L | 2.33 ± 0.10 |
| Phosphate, mmol/L | 1.19 ± 0.21 |
| UACR, mg/mmol | 2.75 (0.90, 8.63) |
| Uric acid, mmol/L | 416.2 ± 90.4 |
| HbA1c | 6.20 (5.80, 6.80) |
| Lipid profile, mmol/L  Total  LDL-C  HDL-C  Triglycerides | 4.78 ± 1.04  2.78 ± 0.84  1.24 (1.13, 1.44)  1.54 (1.09, 2.08) |

Numerical variables are presented as the mean ± standard deviation or median (IQR), categorical variables are defined as absolute numbers and their percentages.

ESS: Epworth Sleepiness Scale; AHI: apnea hypopnea index; OSA: obstructive sleep apnea; SBP: systolic blood pressure; DBP: diastolic blood pressure; BMI: body mass index; 25(OH)D: 25-hydroxyvitamin D; iPTH: intact parathyroid hormone; UACR: urine albumin creatinine ratio; IQR: interquartile range
